# Targeting bias to improve maternal care and outcomes for Black women in the USA

**DOI:** 10.1016/j.eclinm.2020.100568

**Published:** 2020-10-03

**Authors:** Yousof Omeish, Samantha Kiernan

**Affiliations:** a Yale University, New Haven, CT, United States; b Council on Foreign Relations, Washington, DC, United States

Black women in the USA face significant inequities in maternal mortality and morbidity. They die from pregnancy-related deaths at a rate three to four times higher than white women, [1] and are more likely to experience severe maternal morbidity (SMM), life-threatening complications caused or exacerbated by pregnancy, in the antepartum, intrapartum, and postpartum periods. [2] These inequities not only stem from socioeconomic factors affecting access to and quality of care, but are also linked to the stresses of racism—individual and institutional—and their long-term physiological implications. [3] Additionally, implicit and explicit biases in health care can influence whether Black women attend postpartum visits and could, in part, explain these racial disparities. [4,5]

However, there is hope—and an imperative to address these inequities—in that over 60% of pregnancy-related deaths in the USA are preventable. [1] Current legislation such as the *Mothers and Newborns Success Act*, introduced in the US Senate, is investing in important research and funding programs improving maternal care access. The bill supports the US Centers for Disease Control and Prevention's Levels of Care Assessment Tool, which analyzes the level of maternal care in hospitals to inform how they can better meet the needs of communities they serve. It also creates a National Maternal Health Research Network for clinical, epidemiological, and community-based research on maternal mortality, SMM, and the systemic issues that drive racial inequities. For postpartum care, which is especially crucial for women at risk of SMM, the bill establishes a program to identify and implement best practices. [6]

Yet, research on racial bias in clinical settings and numerous stories of Black women experiencing complications after childbirth despite having access to quality care make clear that eliminating the racial inequities of maternal mortality and SMM requires more than just addressing care access. They necessitate a deep look into how implicit and explicit biases in health care put the lives of Black women at risk. The examples of two Black women, Dr. Shalon Irving, a highly educated epidemiologist, and Serena Williams, the famed tennis star, show that these issues are tied to biases. [7] Both women died or nearly died from pregnancy-related complications despite showing symptoms of complications they were at high risk for and reporting these symptoms to health care providers. Indeed, evidence demonstrates that racial inequities transcend education level and wealth. In New York City, Black women with college educations or higher experienced SMM at a rate of 333•0 per 10,000 deliveries as compared to 137•7 per 10,000 deliveries for white women who did not complete high school. [8] These data, in addition to well-documented biases that pervade health care, [9] illustrate the need for solutions tackling implicit bias in clinical training and practice to reduce inequities. Two solutions are actionable and have shown promise.

First, clinical checklists prompting providers to act—through screenings, surveillance, or interventions—if Black women report, show, or are at high risk for symptoms that often portend health emergencies would ensure Black women receive care at hospitals that adheres to particular standards, limiting room for bias. Addressing potential health emergencies through standardized surveillance and protocols that require thorough examination of reported signs or symptoms can help eliminate delayed or overlooked diagnosis and treatment, areas where implicit biases undermine Black women's concerns. Quality improvement initiatives in maternal care have effectively reduced mortality, [3,10] so should similar efforts designed to rid bias from the care Black women receive.

Second, expanding implicit bias training and education in clinical settings is critical to promote awareness of how bias affects care and puts Black women's lives at risk. In many ways, racism is embedded in medical education and practice, and racialized constructions of pain tolerance, for example, affect how, when, and whether pain is treated in Black patients—with direct implications in maternal care. Far too often, these perceptions influence how soon Black women receive needed care; dispelling them and educating providers on how they affect care is crucial.

Racial disparities in health care are systemic at their core, and they require truly comprehensive approaches. In the cases of maternal mortality and SMM, measures reducing barriers to quality care access and confronting structural factors that wear on the day-to-day lives of Black women are essential. Though we least expect racial inequities to be exacerbated in health systems and clinical settings, they nonetheless are. Eliminating inequities and ensuring the health of Black women therefore demand targeting the implicit and explicit racial biases in these settings that endanger their lives.

## Contributors

YO authored the manuscript. SK provided editing support.

## Declaration of Competing Interest

None
